# Modulating CRISPR-Cas Genome Editing Using Guide-Complementary DNA Oligonucleotides

**DOI:** 10.1089/crispr.2022.0011

**Published:** 2022-08-12

**Authors:** Thomas Swartjes, Peng Shang, Dennis T.M. van den Berg, Tim Künne, Niels Geijsen, Stan J.J. Brouns, John van der Oost, Raymond H.J. Staals, Richard A. Notebaart

**Affiliations:** ^1^ Laboratory of Microbiology, Wageningen University and Research, Wageningen, The Netherlands; Delft, The Netherlands.; ^2^ Department of Anatomy and Embryology, Leiden University Medical Centre, Leiden, The Netherlands; Delft, The Netherlands.; ^3^ Food Microbiology, Wageningen University and Research, Wageningen, The Netherlands; Delft, The Netherlands.; ^4^ Department of Bionanoscience, Delft University of Technology, Delft, The Netherlands; and Delft, The Netherlands.; ^5^ Kavli Institute of Nanoscience, Delft, The Netherlands.

## Abstract

Clustered regularly interspaced short palindromic repeats and CRISPR-associated proteins (CRISPR-Cas) has revolutionized genome editing and has great potential for many applications, such as correcting human genetic disorders. To increase the safety of genome editing applications, CRISPR-Cas may benefit from strict control over Cas enzyme activity. Previously, anti-CRISPR proteins and designed oligonucleotides have been proposed to modulate CRISPR-Cas activity. In this study, we report on the potential of guide-complementary DNA oligonucleotides as controlled inhibitors of Cas9 ribonucleoprotein complexes. First, we show that DNA oligonucleotides inhibit Cas9 activity in human cells, reducing both on- and off-target cleavage. We then used *in vitro* assays to better understand how inhibition is achieved and under which conditions. Two factors were found to be important for robust inhibition: the length of the complementary region and the presence of a protospacer adjacent motif-loop on the inhibitor. We conclude that DNA oligonucleotides can be used to effectively inhibit Cas9 activity both *ex vivo* and *in vitro*.

## Introduction

Clustered regularly interspaced short palindromic repeats and CRISPR-associated proteins (CRISPR-Cas) systems provide prokaryotes with adaptive immunity against mobile genetic elements.^1,2^ Similar to other CRISPR-Cas systems, the Cas9 nuclease from *Streptococcus pyogenes* (SpCas9) mediates double-stranded cleavage of the target DNA.^3,4^ The Cas protein binds a guide RNA (gRNA) to form a ribonucleoprotein (RNP) complex, which interrogates the DNA to find a DNA sequence complementary to the gRNA (protospacer).^5^ To this end, the RNP complex binds DNA sequences with a protospacer adjacent motif (PAM) and causes initial unwinding of the adjacent DNA bases.^3–8^ If these bases are complementary to the gRNA, DNA unwinding proceeds along the RNA until a stable R-loop is formed.^5,9^ Lastly, both DNA strands are cleaved, resulting in a double-strand break (DSB) in the target DNA.^3,4^

Cas9 and other Cas nucleases are used for genome editing by introducing DSBs at specific DNA sequences. One possibility is that the genomic edits occur independently of the Cas nuclease, as is generally assumed to take place in most prokaryotes. The nuclease would then be used as a counter-selection system to select against non-edited versions of the target site by introducing DSBs.^10^ Alternatively, in many eukaryotes, for example, the Cas nuclease might first introduce a DSB, which then induces local DNA repair.^11^ The two most common repair mechanisms that act on DSBs are homology directed repair (HDR) and non-homologous end joining (NHEJ).^12^ HDR uses a homologous repair template to fix the DSB according to the template sequence.^12^ NHEJ resolves the double-stranded break without the need for a repair template, which often results in insertions or deletions (indels) at the site of the DSB formation.^13^

For genome editing applications of CRISPR-Cas, it is crucial that the Cas nuclease introduces a DSB at the sequence of interest with sufficient efficacy. However, Cas nucleases were found to also create DSBs at sequences with imperfect complementarity to the gRNA.^14–18^ It is essential to prevent genetic changes at such off-target sites, especially for therapeutic genome editing. In addition, strict control over Cas9 activity might be used to confine DSB formation to the desired cells in a limited time frame.

In nature, Cas enzyme activity can be inhibited by phage-encoded anti-CRISPR (ACR) proteins that act on different stages of CRISPR-Cas-based immunity.^19–21^ The ACRs that were found to inhibit Cas nuclease activity have been reported to be useful for controlling CRISPR-Cas-based genome editing.^21–26^ Aside from these naturally occurring ACRs, several other strategies have been devised to control Cas enzyme activity at the level of transcription,^27–29^ translation,^30–32^ protein state,^33–47^ and gRNA.^14,48^

In addition, single-stranded DNA or RNA molecules can be designed to inhibit Cas nucleases. Such oligonucleotide-based inhibitors provide several advantages compared with natural ACR proteins. DNA oligos are inexpensive, can be rapidly manufactured, and could provide a systematic way to inhibit different Cas nucleases, whereas the use of ACRs is dependent on the compatibility with the Cas nuclease of choice.

Oligo-based inhibitors have been shown to work *in vitro* and in human cell cultures for Cas9 and Cas12a.^49–51^ Potent inhibition of Cas9 was observed with RNA-DNA hybrids or chemically modified DNA inhibitors that interact with the repeat sequence of the gRNA or with the PAM-interacting domain of Cas9.^50^ In addition, truncated gRNA designs have been shown to allow dsDNA binding, but not cleavage by Cas9.^52,53^ Such truncated gRNAs were used to specifically direct non-cleaving Cas9 RNPs to off-target sequences, thereby preventing active Cas9 RNP from binding these off-target sites.^51^ Lastly, DNA oligos with phosphorothioate linkages displayed strong inhibition of Cas12a activity, independent of the nucleotide sequence of the inhibitor used.^49^

In the current study, we assessed whether SpCas9 could be inhibited with guide-complementary DNA oligonucleotides without any chemical modifications. To this end, we designed and tested different oligo-based inhibitors complementary to the spacer-derived part of the gRNA (these oligos thus have the same sequence as the PAM-proximal part of the protospacer). We also investigated the effect of extending the inhibitors with a double-stranded PAM sequence. We show that various designs provide strong, sequence-dependent inhibition of Cas9 *in vitro* and *ex vivo*.

In addition, we investigated the effect of oligo-based Cas9 inhibition on off-target sites in the context of genome editing. We found that while inhibition reduces both on- and off-target activity of Cas9, the presence of specific oligo designs results in slightly increased specificity. By comparing the inhibitor results with a Cas9 titration, we conclude that the increased specificity is a general consequence of lowering overall Cas9 activity.

Lastly, we studied which mechanisms lead to the observed inhibition. We conclude that the effect of the tested inhibitors is dependent both on the length of the oligo-based inhibitors and on the presence of a PAM-loop. Their relative importance is strongly affected by the speed at which Cas9 cleaves the targeted DNA.

## Materials and Methods

### Cell line and cell culture

The chronic myelogenous leukemia (CML) cell line used is a rediploidized derivative of the HAP1 cell line, which was a kind gift of Dr. Thijn Brummelkamp.^54^ CML cells were cultured in Iscove's modified Dulbecco's medium (Gibco), supplemented with 10% fetal bovine serum and 1% penicillin/streptomycin. Cells were grown at 37°C in a humidified atmosphere containing 5% CO_2_.

### Cas9 protein, gRNA, and inhibitors used *ex vivo*

Recombinant *S. pyogenes* Cas9 (variant with multiple nuclear localization signals)^55^ and high-fidelity protein variants (SpCas9-HF1^56^ or evoCas9)^57^ were provided by Geijsen lab through Divvly (https://divvly.com/geijsenlab). gRNAs used are synthetic gRNAs, which contain the target-specific CRISPR RNA (crRNA) and the scaffold trans-activating CRISPR RNA (tracrRNA) (IDT). The crRNA and tracrRNA were dissolved in the nuclease-free Duplex buffer (IDT) to reach the concentration of 200 μM. Equal volumes of dissolved crRNA and tracrRNA were mixed and annealed by heating for 5 min at 95°C and cooling down at room temperature. The oligo-based inhibitors used were unmodified single-stranded DNA oligos synthesized by IDT (IDT). Each of such oligos was dissolved in nuclease-free water to reach the concentration of 75 μM.

### Induced transduction by osmocytosis and propanebetaine

The recombinant Cas9 proteins, gRNAs, and oligo-based inhibitors were simultaneously transduced into CML cells by using the induced transduction by the osmocytosis and propanebetaine (iTOP) method as described previously.^58^ One day before the transduction, CML cells were plated at 18,000 cells/well in the Matrigel-coated wells on 96-well plates, such that on the day of transduction, cells would reach about 70–80% confluence. Next day, for each well of the 96-well plate, 50 μL of iTOP mixture that contains 20 μL of transduction supplement (Opti-MEM media (Thermo Fisher Scientific) supplemented with 542 mM NaCl, 333 mM gamma-aminobutyric acid, 1.67 × N2, 1.67 × B27, 1.67 × non-essential amino acids, 3.3 mM glutamine, 167 ng/mL bovine fibroblast growth factor-basic, and 84 ng/mL epidermal growth factor), 10 μL of Cas9 protein (75 μM), 7.5 μL of gRNA (100 μM), 10 μL of oligo-based inhibitors, and the excess volume of nuclease-free water to reach the 50 μL total volume was prepared.

For the no-protein control, 10 μL of protein storage buffer was used instead of the CRISPR nuclease protein; and for the no-guide control, an equal volume of nuclease-free water was used to replace the gRNA or oligo-based inhibitors. The 50 μL iTOP mixture was added onto the cells immediately after the culture medium was removed. The plate then was incubated in a cell culture incubator for 45 min, after which the iTOP mixture was gently removed and exchanged for 250 μL of regular culture medium.

### Isolation of genomic DNA in 96-well cell culture plates

Genomic DNA of each transduced cell sample was purified by direct in-plate cell lysis and DNA isolation according to a previously published protocol.^59^ Briefly, after aspirating culture media and adding 50 μL of lysis buffer (10 mM Tris HCl pH 7.6, 10 mM ethylenediaminetetraacetic acid (EDTA), 100 mM NaCl, 0.5% N-lauroyl sarcosine sodium, 50 μg/mL RNase A, and 100 μg/mL proteinase K) to each well, the sealed plate was incubated at 55°C overnight; added 100 μL of ice-cold NaCl-saturated ethanol (for 100 mL 100% ethanol, add 1.5 mL of 5 M NaCl) to each well; allowed the plate to stand still for 4 h at room temperature to precipitate the DNA; washed the precipitated DNA with 75% ethanol for two times and let the plate air dry; DNA in each well was dissolved in 50 μL of Tris-EDTA buffer.

### Deep-sequencing preparations

The regions of interest (Supplementary Table S1) were polymerase chain reaction (PCR) amplified (Supplementary Table S2) from the extracted DNA using Q5 high-fidelity DNA polymerase (New England Biolabs). Of the primers used in the PCRs (Supplementary Table S3), the forward primers contained 5nt sequencing barcodes. All 576 amplifications were verified using 20 g/L agarose gel electrophoresis. Equal volumes of the PCR products were pooled with samples from the same locus, but with unique barcodes. These 36 pools were then purified (Zymo Research Z4004) and quantified using Qubit dsDNA BR (Thermo Fisher Scientific). Equal amounts of DNA from the samples were then pooled further to provide six pools where each sample contains a unique combination of sequencing barcode and amplified region. These samples were then sent to BaseClear B.V. for quality control, index PCR, and sequencing on the NovaSeq 6000 for paired-end 150nt-long reads.

The raw sequencing results are available on the NCBI Sequence Read Archive under BioProject PRJNA796802.

### Analysis of deep-sequencing results

To analyze the deep-sequencing data, the paired-end reads were programmatically merged using *seqprep.*^60^ Then, the reads were filtered out that do not match the expected pattern of starting with a barcode followed by a forward primer annealing part, and ending with the associated reverse primer annealing part. We then split the reads by their barcodes and mapped the reads to the human genome (hg38) using *bowtie2*.^61,62^ The resulting alignments were then, using *samtools*,^63,64^ sorted, indexed, and split by the locus of interest where they align to the human genome. A separate script was used to score the amount of reads with insertions or deletions based on the concise idiosyncratic gapped alignment report (CIGAR)-strings from the alignment files. Another script was used to—for each indel—record its position and length. The used scripts were created in-house and will be made available upon reasonable request.

### Calculation of specificity

*Percentage specificity* was calculated by dividing the *percentage reads with indels* of the on-target locus by the sum of *percentage reads with indels* across all three loci (on-target, off-target1, and off-target2). The resulting value answers the question “What percentage of the observed indels happen on-target?” corrected for the total number of reads at each locus.

### DNA cleavage assays

The relative inhibitory effect of the oligo-based inhibitors was determined using *S. pyogenes* Cas9 (New England Biolabs), Alt-R CRISPR-Cas9 single guide RNA (sgRNA) (IDT), EDTA (Merck), and proteinase K (New England Biolabs). The substrate was obtained by PCR (Supplementary Table S4) on CML cell-derived genomic DNA using Q5 DNA polymerase (New England Biolabs) and purified on a 10 g/L agarose gel using the Wizard SV Gel and PCR Clean-Up system (Promega). All mixtures were made in 1 × NEBuffer 3.1 on ice with final concentrations of 80 nM Cas9, 320 nM sgRNA, 8 nM substrate, and 1000 nM oligo. For the preincubation assays, Cas9 and sgRNA were mixed and preincubated for 20 min at 25°C, and in the meantime, the substrate and oligo mixes were made. Equal volumes of RNP and substrate+oligo mixes were combined and incubated at 37°C for 30 min.

### Visualization and quantification of DNA cleavage

Reactions were stopped by placing them on ice and immediately adding 0.05 V of 0.5 M EDTA pH = 8.0 and 0.05 V of proteinase K. Time point 0 samples were made by immediately stopping the reactions and storing them on ice during the incubation. After stopping, samples were incubated at room temperature for 10 min and then the TriTrack loading dye (Thermo Fisher Scientific) was added to a final concentration of 1 × and 12 μL was loaded on a 10 g/L agarose gel. Gels were run for 45 min at 100 V in 1 × Tris-acetate-EDTA buffer using SYBRSafe (Thermo Fisher Scientific) as the staining agent. Bands were visualized using the UVITEC Alliance (UVITEC) and quantified using the Image Lab software (Bio-Rad) version 6.0.1 build 34. Relative quantity of the remaining substrate was determined by setting the time point 0 samples of each as 1 and measuring the relative intensity. % Digestion was calculated as (1 − Irel) × 100%. Assays were performed three times independently for each substrate.

For the non-preincubated *in vitro* assay, the concentrations of all compounds were the same, but the mixtures were substrate+Cas9 and oligo+sgRNA and they were combined on ice before incubation at 37°C.

### Time-shift or time-lapse assay

For the time-lapse assay, the same compounds and concentrations were used as for the *in vitro* assays, but the preformed RNP and inhibitor were preincubated in 30-s steps before addition of the substrate. Therefore, three premixes were made, Cas9 and gRNA (RNP), oligo, and substrate were diluted in 1 × NEBuffer 3.1. The RNP complexes were preformed by incubation at 25°C for 20 min, and 5 μL of oligo dilution was put in 1.5-mL tubes (or buffer in the case of the “no inhibitor” control). All samples were put in a 37°C heat block, and at different time points, 10 μL of the RNP or 5 μL of the substrate was added, and so, all samples had a digestion time of 30 min. Reactions were stopped and samples analyzed on gel as described above.

### Electrophoresis mobility shift assay

Final concentrations of the compounds for the electrophoresis mobility shift assay (EMSA) were 170 nM Cas9, 170 nM sgRNA, and 500 nM oligo. Compounds were added together and preincubated at 37°C for 15 min, after which a buffer or the tested compound was added and the samples were incubated at 37°C for 15 min. TriTrack loading dye was added and 5 μL was loaded on a 5% polyacrylamide gel in 0.5 × Tris-Borate (TB) buffer (45 mM Tris, 45 mM boric acid) and run at 15 mA for 40 min. The gel was stained with SYBRGold (Thermo Fisher Scientific) for 5 min, destained in 0.5 × TB buffer for 10 min, and visualized using the UVITEC Alliance (UVITEC).

### Data visualization and statistics

The data presented in this study were visualized using the *datavis.ipynb* Jupyter Notebook file. In addition to *Jupyter Notebook*,^65^ we used *Python3*,^66^ *numpy*,^67^ *pandas*,^68^ *matplotlib*,^69^ and *seaborn*.^70^ In cases where results were quantified, triplicates were used for each condition. Where statistics were indicated, those were done by conducting a Shapiro–Wilk test for normality, a Levene's test for equal variance, a one-way analysis of variance, and lastly a Tukey's range *post hoc* test. For these statistics, we used *SciPy*^71^ and *statsmodels*^72^ in addition to the programs and packages used for the data visualization.

## Results

**FIG. 1.**
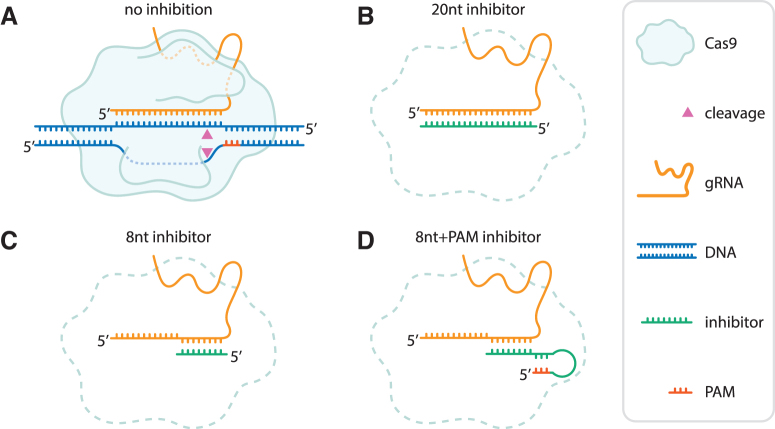
Design of guide-complementary oligo-based inhibitors. Schematic representation of a Cas9 nuclease, gRNA (here displayed as a sgRNA) with and without oligo-based inhibitors. **(A)** Without inhibitors, the RNP binds the target sequence in genomic DNA, forms an R-loop, and proceeds to cleave both strands of the DNA (triangles). **(B)** 20nt inhibitor. **(C)** 8nt inhibitor. **(D)** 8nt+PAM inhibitor. For all nucleic acids, 5′ ends are indicated. gRNA, guide RNA; PAM, protospacer adjacent motif; RNP, ribonucleoprotein; sgRNA, single gRNA.

To assess whether guide-complementary oligonucleotides could work as guide-specific inhibitors of Cas nucleases, we designed guide-complementary DNA oligos (hereafter referred to as “inhibitors”) of 8 and 20 nucleotides (nt) (Fig. 1). The inhibitors are complementary to the PAM-proximal, spacer-derived part of the gRNA for SpCas9 (Fig. 1B, C). We also designed oligos with a 5′-extension intended to loop and fold back onto itself, creating a double-stranded PAM (Fig. 1D).

### DNA oligo-based inhibitors reduce Cas9 activity in human cells

We set out to test the effects of these DNA oligo-based inhibitors in a genome editing context. To that end, we delivered purified SpCas9 protein, gRNA, and the inhibitors to CML cells using iTOP.^58^ We analyzed editing of two endogenous gene loci, EMX1-1 and FANCF-2, under all test conditions. iTOP-transduced cells were allowed to grow to 90% of confluency, and we then extracted the genomic DNA from the cells. From the genomic DNA, we amplified six regions of interest: two on target sites (EMX1-1 and FANCF-2) and two, previously identified,^73,74^ prominent off-target sites for each target (Supplementary Table S1). We performed deep-sequencing on the amplicons to assess the frequency of indels resulting from NHEJ.

**FIG. 2.**
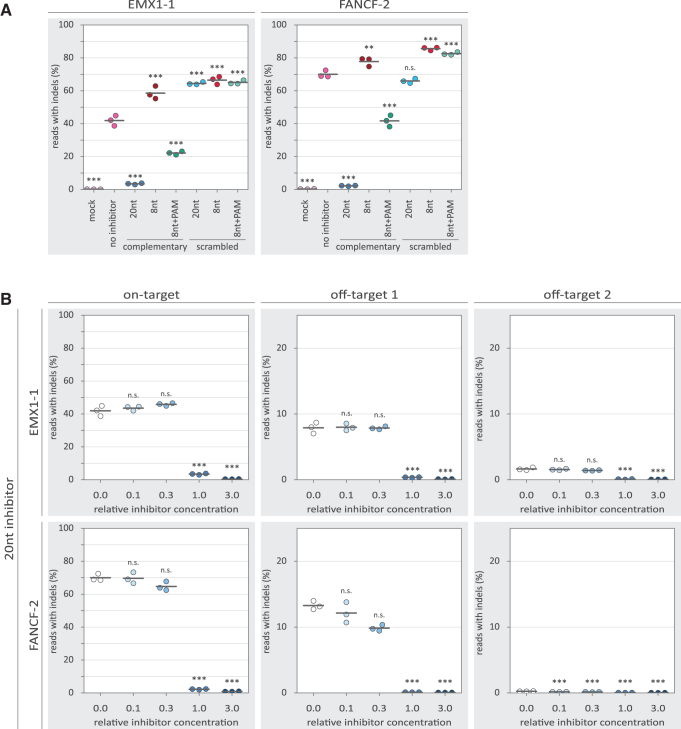
Inhibition of Cas9 by guide-complementary DNA oligos. Individual replicates are displayed as colored dots, while the horizontal black lines show the mean of the three replicates. *p*-Values were calculated with a one-way ANOVA and subsequent Tukey's test. “n.s.” not significant; **p-value <0.01; ***p-value <0.001. **(A)** Percentage of reads containing indels for on-target loci of EMX1-1 and FANCF-2. The “mock” condition constitutes a control where the induced transduction by osmocytosis and propanebetaine method was used without delivering Cas9, gRNA, and inhibitor. All conditions except the “mock” used SpCas9, gRNA, and—except for “no inhibitor”—a DNA oligo design, as detailed in Figure 1. The DNA oligos were delivered at a molar concentration equal to the concentration of Cas9 and gRNA. *p*-Values are displayed for the “mock” condition compared with each other condition. **(B)** Percentage of reads containing indels for on- and off-target loci of EMX1-1 and FANCF-2 for different concentrations of the 20nt inhibitor. The “relative inhibitor concentration” indicates the molar concentration of the inhibitor relative to Cas9 and gRNA. A “relative inhibitor concentration” of 1.0 means that the used molar concentration of the inhibitor is equal to that of Cas9 and gRNA. For off-target loci, the *y*-axis is adjusted because relatively few indels contained indels in these conditions. *p*-Values are displayed for the 0.0 relative oligo concentration compared with each other condition. ANOVA, analysis of variance.

Without inhibitors, iTOP-delivered Cas9 RNP achieved roughly 40% and 70% on-target indel frequencies on EMX1-1 and FANCF-2 loci, respectively (Fig. 2A). Addition of the 20nt inhibitor—in equimolar ratio to the Cas9 RNP—strongly reduces indel frequency (to less than 5%), indicating that Cas9-induced DSB formation is repressed. Repression is also observed with the 8nt+PAM inhibitor, but to a lesser extent (between 20% and 50%). The 8nt inhibitor, however, unexpectedly showed increased indel frequencies rather than inhibition. The same was observed for most of the sequence-scrambled versions of the inhibitors, which were included as a control for sequence specificity. We did not observe a substantial effect of the tested inhibitors on the positions and lengths of the indels (Supplementary Figs. S1 and S2). Together, this shows that both the 20nt and 8nt+PAM inhibitors repress Cas9 activity *ex vivo.*

### Inhibition affects on- and off-target activity of Cas9 to different extents

**FIG. 3.**
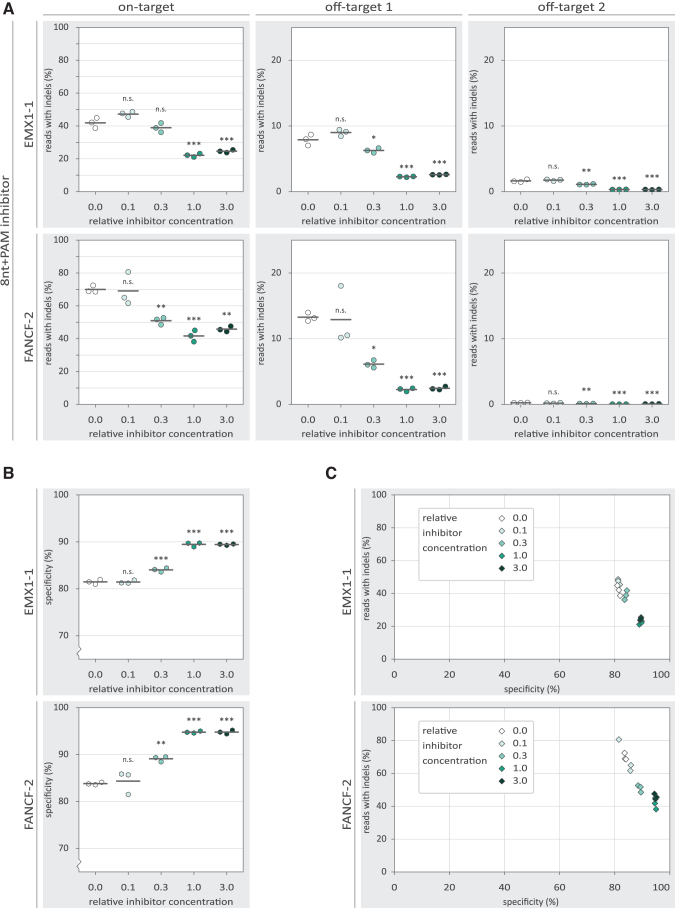
The effects of 8nt+PAM inhibitor concentration on the specificity of Cas9-mediated NHEJ. *p*-Values were calculated with a one-way ANOVA and subsequent Tukey's test. *p*-Values are displayed for the 0.0 relative oligo concentration compared with each other condition. “n.s.” not significant; *p-value <0.05; **p-value <0.01; ***p-value <0.001. **(A)** Percentage of reads containing indels for on- and off-target loci of EMX1-1 and FANCF-2 for different concentrations of the 8nt+PAM inhibitor. The “relative inhibitor concentration” indicates the molar concentration of the inhibitor relative to Cas9 and gRNA. A “relative inhibitor concentration” of 1.0 means that the used molar concentration of the inhibitor is equal to that of Cas9 and gRNA. For off-target loci, the *y*-axis is adjusted because relatively few indels contained indels in these conditions. **(B)** Percentage specificity for on-target loci of EMX1-1 and FANCF-2 for different concentrations of the 8nt+PAM inhibitor. The *y*-axis starts at 70% specificity to better visualize the differences between conditions. The individual replicates are displayed as colored hexagons, while the horizontal black lines show the mean of the three replicates. **(C)** Comparison of percentage reads with indels and percentage specificity for different concentrations of the 8nt+PAM inhibitor. For each inhibitor concentration, individual replicates are displayed as diamonds with the same color. NHEJ, non-homologous end joining.

To characterize the observed inhibition in more detail, we tested the inhibitors in a concentration gradient. Substantial inhibition by the 20nt inhibitor is only observed at and above the equimolar ratio to the Cas9 RNP (Fig. 2B). For the 8nt+PAM inhibitors, a more gradual dose–response is observed (Fig. 3A), with low concentrations seemingly providing low levels of inhibition. However, even at the highest concentration, the 8nt+PAM inhibitors are not able to reduce indel frequencies to the same extent as was achieved with the 20nt inhibitors. At the investigated off-target loci, we observed reduced indel frequencies with the 20nt and 8nt+PAM inhibitors, following a similar dose–response as was seen on-target.

The 8nt+PAM oligo displays inhibition both on- and off-target, but neither is completely abolished. This raises the question to what extent the ratio between on- and off-targeting is affected by the inhibitors. We calculated a specificity score by dividing the on-target indel frequency by the sum of the on- and off-target indel frequencies. This specificity score signifies the percentage of indels that were mapped to the on-target locus, corrected for the total number of reads mapped to each locus. We found that the higher concentrations of the 8nt+PAM inhibitor slightly increase the specificity score (Fig. 3B). However, it is important to realize that at the same inhibitor concentrations, on-target activity is substantially reduced (Fig. 3B, C).

For the 20nt inhibitor, we observed increased specificity only in conditions in which barely any on-target activity is left (Supplementary Fig. S3). We also tested variant 20nt inhibitors (“off1–20nt” and “off2–20nt”) based on the off-target sequences (Supplementary Table S5). These off-target 20nt inhibitors did not show substantial differences in specificity or activity compared with the on-target 20nt inhibitors (Supplementary Fig. S3). This suggests that it is not possible with the inhibitors reported here to selectively inhibit only one of the sites that are targeted with the same gRNA.

**FIG. 4.**
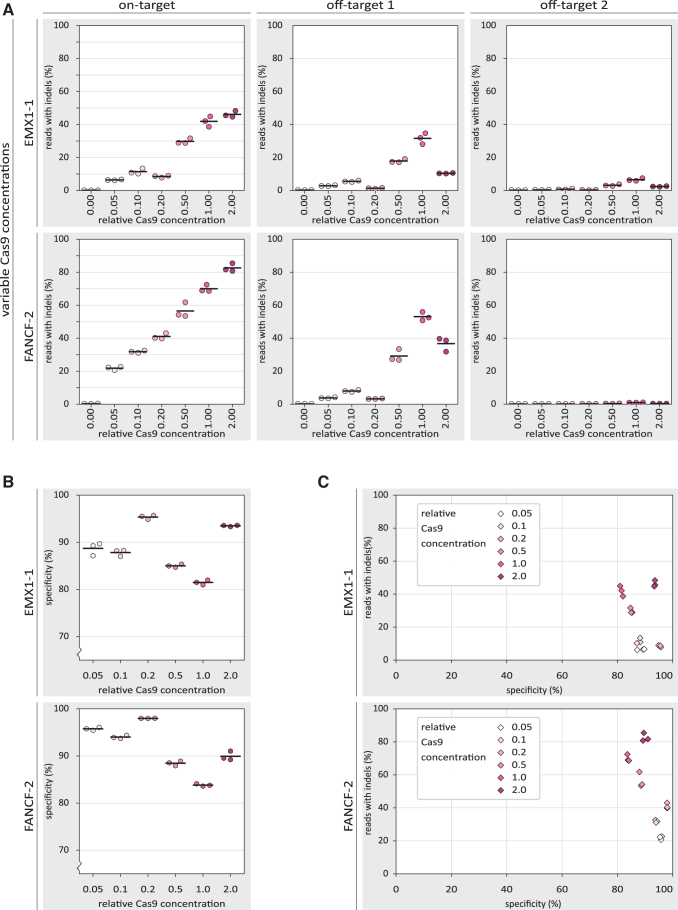
The effects of Cas9 concentration on specificity of Cas9-mediated NHEJ. **(A)** Percentage of reads containing indels for on- and off-target loci of EMX1-1 and FANCF-2 for different concentrations of SpCas9 RNP in the absence of DNA oligo-based inhibitors. The individual replicates are displayed as colored dots, while the horizontal black lines show the mean of the three replicates. The concentration 0.00 is the “mock” condition in Figure 2A. **(B)** Percentage specificity for on-target loci of EMX1-1 and FANCF-2 for different concentrations of Cas9 RNP. The *y*-axis starts at 70% specificity to better visualize the differences between conditions. The individual replicates are displayed as colored hexagons, while the horizontal black lines show the mean of the three replicates. The 0.00 Cas9 RNP concentration is not included here because the specificity values make no sense if there are virtually no indels at all. **(C)** Comparison of *percentage reads with indels* and *percentage specificity* for different concentrations of the Cas9 RNP. For each inhibitor concentration, individual replicates are displayed as diamonds with the same color. Again, the 0.00 Cas9 RNP concentration is not included here.

We next asked whether the observed increase in specificity may be the result of a decreased subpopulation of active Cas9 RNP complexes through inhibition with DNA oligos. To address this, we looked at a range of Cas9 RNP concentrations to see how they affect on-target activity and specificity. At both on-target loci, we found that increasing RNP concentrations lead to higher indel frequencies (Fig. 4A). Strikingly, at the off-target sites, the highest Cas9 RNP concentration yielded relatively low indel frequencies.

The resulting specificity scores are highest at relatively low RNP concentrations, and drop to a minimum at high concentrations. At the highest concentration, however, specificity increases again, due to the unexpected decrease in off-target indels at that RNP concentration (Fig. 4B). When plotting both the on-target activity and specificity, among the intermediate Cas9 concentrations, we observed a similar trade-off between activity and specificity as was observed with the 8nt+PAM inhibitor (Fig. 4C). The lowest and highest Cas9 RNP concentrations do not follow this pattern, however.

### Inhibitors with a PAM-loop efficiently inhibit preformed Cas9 RNP *in vitro*

So far, we have seen that the 20nt inhibitor provides the most potent inhibition in our *ex vivo* setup, almost abolishing on- and off-target indel formation. The 8nt+PAM version displayed less inhibition, but shows some level of inhibition already at low concentrations. To elucidate why the 20nt and 8nt+PAM inhibitors display different dose–responses, we designed an experimental approach to better understand the mechanism by which the inhibitors function. To that end we used *in vitro* cleavage assays, which provide more experimental control than the *ex vivo* setup.

**FIG. 5.**
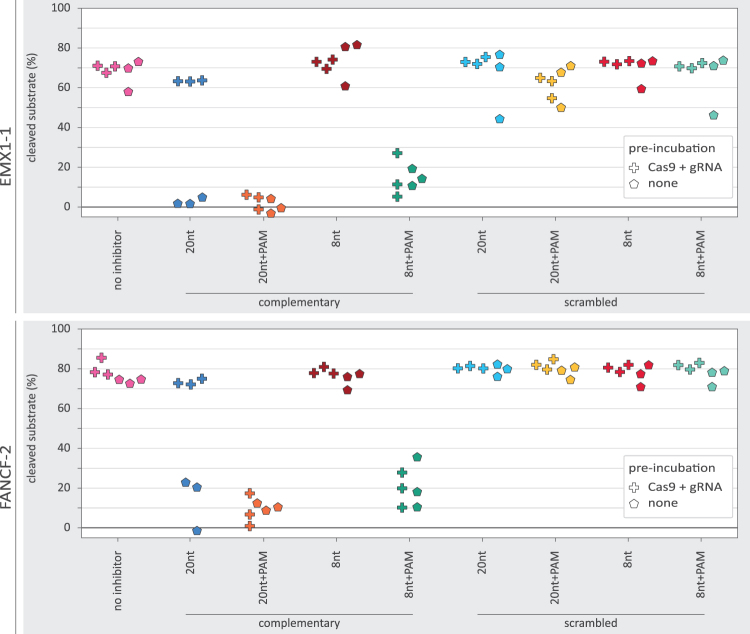
*In vitro* DNA cleavage in the presence of various oligo-based inhibitor designs. The percentage of DNA substrate cleaved as measured from band intensity on agarose gel. The plus-markers show the observed percentage of DNA cleavage when Cas9 and gRNA were preincubated. The pentagon-markers represent replicates without preincubation. For EMX1-1 with oligo and gRNA preincubation, the scrambled oligo designs each have a single data point that displays relatively poor cleavage. These low data points are all derived from a single batch of substrate DNA purified from agarose gel, different from the other data points.

In preparation for the *in vitro* experiments, we performed PCRs of the EMX1-1 and FANCF-2 loci to produce the cleavage substrate DNA (Supplementary Table S4). First, we incubated the purified Cas9 protein with the appropriate gRNA (20 min preincubation at 25°C: Cas9+gRNA). Second, we added the target dsDNA and the oligo-based inhibitors to the Cas9 and guide and incubated the reactions to allow cleavage of the DNA (30 min 37°C). We then used agarose gel electrophoresis to assess how much of the substrate DNA had been cleaved. We found that the preincubated Cas9 and gRNA could efficiently digest the substrate DNA (Fig. 5). However, addition of the 8nt+PAM inhibitor substantially reduced DNA cleavage.

In agreement with the aforementioned *ex vivo* analyses, we did not observe an effect from the 8nt inhibitor, nor with the sequence scrambled inhibitors. Unexpectedly, the 20nt inhibitor also did not show a clear reduction in DNA cleavage, despite strongly inhibiting indel formation *ex vivo*.

To better understand the differences between *ex vivo* and *in vitro* outcomes, we repeated the *in vitro* cleavage assays, but this time without the initial incubation of Cas9 with gRNA (preincubation: none). In addition, we now included a 20nt+PAM inhibitor design in both experimental setups to see whether the PAM loop could rescue the performance of the 20nt inhibitor. We now found that the 20nt inhibitor strongly inhibited DNA cleavage (Fig. 5). The 20nt+PAM inhibitor displayed strong inhibition in both experimental setups. This shows that inhibition without a PAM-loop is dependent on the order in which the inhibitor, Cas9, and gRNA are mixed. In contrast, the PAM-loop enables inhibition regardless of the mixing order. The 8nt inhibitor without PAM-loop did not show any *in vitro* inhibition regardless of which experimental conditions were used.

### Inhibitors with a PAM-loop require less time to inhibit Cas9 RNP

**FIG. 6.**
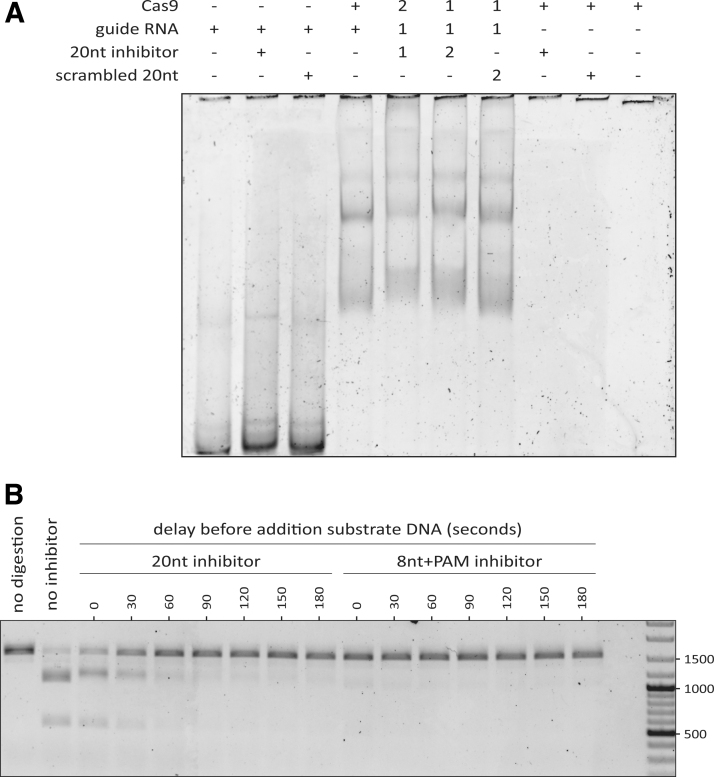
Timing-dependent inhibition of Cas9 by oligo-based inhibitors. **(A)** Electrophoretic mobility shift assay on 5% polyacrylamide gel, stained with SYBR Gold for nucleic acid visualization. Components with a “+” sign were present, whereas those with a “-”sign were not. A “1” indicates that these components were initially mixed. In contrast, the components marked with “2” were added after 15 min of initial incubation. The black lines at the top of the image correspond to the bottom of the wells of the gel. **(B)** Time-lapse assay where the 20nt or 8nt+PAM inhibitors were added to preformed RNP complexes, then incubated for variable durations (“delays”) before addition of the substrate DNA. The GeneRuler mix ladder was included on the far right and relevant fragment lengths are indicated in base-pairs. The linear substrate DNA is 1500 bp long and cleavage by Cas9 would result in two fragments of lengths 1000 and 500 bp.

We wondered whether the PAM-less 20nt inhibitor would only be able to bind the gRNA when the RNA is not yet bound by Cas9. To assess this, we performed an EMSA to visualize binding between the inhibitor, Cas9, and gRNA in different combinations. From this, we observed a large shift upon adding Cas9 to the gRNA, indicating that the two bind to each other (Fig. 6A). If the 20nt inhibitor is added as well, the bands shift up slightly more, regardless of the order in which the components are mixed. This is not the case with the scrambled 20nt inhibitors, indicating that binding of the inhibitor is sequence-specific. Overall, we conclude that the 20nt inhibitor can bind both preformed RNP complexes and free gRNA. It is therefore likely that the 20nt inhibitor can also inhibit at both stages.

We then asked why—given the EMSA results—we did not observe inhibition with the 20nt inhibitor on preincubated Cas9 and gRNA (Fig. 5). We reasoned that the 20nt inhibitor might require a longer time to bind preformed RNP compared with free gRNA. If that is the case, the 20nt inhibitor would simply not have had enough time to bind (thus inhibiting cleavage of the DNA substrate by Cas9 RNP). We then tested how much time the 20nt inhibitor requires for inhibition of preformed Cas9 RNP. To that end, we first incubated the gRNA and Cas9 to form the RNP. The preformed RNP was then incubated with the 20nt or 8nt+PAM oligo for a variable duration before adding the substrate DNA and allowing 30 min of digestion.

We observed that the 20nt inhibitor requires roughly 1 min to prevent most of the DNA cleavage upon subsequent addition of the substrate DNA (Fig. 6B). Inhibition by the 8nt+PAM design appears to be instantaneous. This explains why we did not observe a substantial reduction in DNA cleavage when the 20nt inhibitor was added simultaneously with the substrate DNA. Taken together, the *in vitro* results presented here show that a PAM-loop affects how the oligo-based inhibitors function. Designs with a PAM-loop can efficiently inhibit DNA cleavage regardless of whether they are added to free gRNA, or to preformed RNP complexes. In contrast, PAM-less designs are effective when added to free gRNA, and require a longer incubation to inhibit preformed RNP complexes.

## Discussion

We have described sequence-dependent inhibition of Cas9 with guide-complementary unmodified DNA oligos. In CML cells, we observed inhibition of on- and off-target NHEJ. Depending on the inhibitor design used, indels could be virtually eliminated. In addition, we used *in vitro* assays to show that a PAM-loop enables robust inhibition by decreasing the time required for inhibition to take place. This PAM-loop design was inspired by another study where DNA oligos were used to create a double-stranded PAM.^75^ In contrast to most previously described Cas-inhibiting oligos,^49,50^ inhibition with the inhibitors described here is dependent on the sequence of the gRNA (Supplementary Fig. S5). Because of their sequence-specificity, these inhibitors theoretically allow modulating Cas9 activity at multiple sites independently.

Indeed, *in vitro* we could independently inhibit cleavage of either EMX1-1 or FANCF-2 DNA substrate (Supplementary Fig. S6). Because the inhibitors seem to act as competitive inhibitors on the Cas9 RNP, this could only work for sites that are targeted by different gRNAs. Using this setup, it would therefore not be possible to exclusively inhibit off-target activity. We found that the sequence-scrambled versions of the oligos (as well as the short [8 nt] complementary oligos) increased Cas9 activity, rather than inhibiting it. These oligos do not inhibit Cas9 likely because if they bind the RNP they would easily be replaced by the target DNA. Concerning the observed increase in Cas9 activity, perhaps the oligos improve the efficiency of Cas9 and/or gRNA delivery through iTOP, similar to how carrier DNA can improve transfection efficiency.^76–78^ Alternatively, the DNA oligos might bind intracellular RNAs, preventing them from inhibiting RNP formation.^79^

We found that the 20nt oligo is the most potent inhibitor tested in CML cells. The 20nt oligo outperforms shorter inhibitors, most likely because it has higher affinity to the gRNA. The PAM-loop is thought to increase affinity, partially rescuing inhibition by shorter oligos. Unexpectedly, the 8nt+PAM consistently showed a slightly different concentration response compared with the 20nt inhibitor (Figs. 2B and 3A). This might be partially explained by assuming that the 20nt inhibitor is cleaved by Cas9, while the 8nt+PAM is not. Indeed, *in vitro* ssDNA cleavage by SpCas9 has been described ^3^ and DNA cleavage requires interactions between the protein and “PAM distal” bases of the DNA,^80^ which are not present in the 8nt or 8nt+PAM inhibitors. This might allow the 8nt+PAM inhibitor to evade cleavage and inhibit Cas9 near-optimally at an equimolar ratio to the RNP.

In cases where inhibition should last, a shorter-than 20nt oligo (perhaps with a PAM-loop to improve affinity) might therefore be preferred. Alternatively, chemical modifications—already proven to yield potent Cas nuclease inhibitors^49,50^—can prevent cleavage of inhibitors.

We found that the DNA oligo-based inhibitors affect both on- and off-targeting by Cas9 and can slightly increase specificity for activity on-target. However, the observed increase in specificity is minor and coincides with a substantial reduction of on-target activity. To put these results into context, we also tested engineered Cas9 variants (SpCas9-HF1^56^ or evoCas9^57^) without inhibitors. We found that these variant Cas9 proteins are mostly more effective at increasing the specificity than the inhibitors reported here (Supplementary Fig. S4). We thus conclude that for increasing the specificity of Cas9, other measures (such as using engineered Cas9 protein variants) might be more suitable.

*In vitro*, the 20nt inhibitor design did not show efficient inhibition when RNPs were precomplexed. Indeed, the PAM-less inhibitors required more time to inhibit preformed RNP compared with the designs with a PAM-loop. *Ex vivo*, the Cas9 protein and gRNA likely entered the cell as free protein and RNA. Therefore, additional time was required to form RNPs, possibly providing the 20nt inhibitor more time for strong inhibition. In addition, in the CML cells, the on-target DNA substrate is presumably at a relatively low concentration compared with the *in vitro* conditions and—once the RNP is formed—the time required for the Cas9 RNP to find its cognate target is much longer. This too would give more time to the 20nt design to establish strong inhibition, possibly explaining the observed differences between the *ex vivo* and *in vitro* experiments.

In an attempt to reveal the molecular basis of the specificity increase, we included conditions without inhibitors present where we varied the concentration of Cas9. The resulting data suggest that the observed increase in specificity could be a general consequence of lowering the concentration of active (cleavage-competent) RNPs, which has previously been described and explained by others.^15,81,82^ Unexpectedly, our highest Cas9 concentration led to increased on-target activity, but reduced off-target activity (Fig. 4A). The mechanism behind this is unknown and requires further research.

## Conclusion

In this study, we tested whether guide-complementary DNA oligos can be used to inhibit Cas9 activity. In CML cells, the most potent inhibition was observed with inhibitors that have the longest complementarity to the gRNA. The *in vitro* experiments showed that a PAM-loop is required for rapid inhibition. Although the inhibitors performed slightly differently *in vitro* compared with *ex vivo*, in both settings, DNA oligo-based inhibitors provided potent inhibition of Cas9 activity. Unmodified DNA oligos are inexpensive and easily manufactured and their design could easily be adapted to other RNA-guided nucleases. Overall, it is concluded that the guide-complementary DNA oligos reported here are promising candidates for sequence-dependent inhibition of Cas9 activity.

## Supplementary Material

Supplemental data

Supplemental data

Supplemental data

Supplemental data

Supplemental data

Supplemental data

Supplemental data

Supplemental data

Supplemental data

Supplemental data

Supplemental data
