# Supplementary material for: Modulating CRISPR-Cas Genome Editing Using Guide-Complementary DNA Oligonucleotides
Source: CRISPR J. 2022 Aug 12;5(4):571–85. doi: 10.1089/crispr.2022.0011 (PMC9419950; doi:10.1089/crispr.2022.0011)
Supplement: Supplemental data [file Suppl_TableS1.docx]

| **Supplementary table 1. Genomic loci.**  The genomic loci that were targeted in the *ex vivo* experiment, and which were amplified to provide DNA substrate for the *in vitro* experiments. For each target we list whether it is included as an on- or off-target site, which on-target site it relates to, the nucleotide sequence, the PAM, the chromosome on which it is located, the coordinate of where on the chromosome it is located, and finally references to sources, based on which we decided to include these loci. | | | | | | |
| --- | --- | --- | --- | --- | --- | --- |
| **on/off** | **on-target** | **sequence** | **PAM** | **chr** | **coordinate** | **refs** |
| on | EMX1-1 | GAGTCCGAGCAGAAGAAGAA | gGG | 2 | 72933853 | ^55^ |
| off1 | EMX1-1 | GAGTTAGAGCAGAAGAAGAA | aGG | 5 | 45358962 | ^55^ |
| off2 | EMX1-1 | GAGTCTAAGCAGAAGAAGAA | gAG | 15 | 43817549 | ^55^ |
| on | FANCF-2 | GCTGCAGAAGGGATTCCATG | aGG | 11 | 22625792 | ^56^ |
| off1 | FANCF-2 | GCTGCAGAAGGGATTCCAAG | gGG | 22 | 36556948 | ^56^ |
| off2 | FANCF-2 | GACGCAGAAGGGACTCCATG | gGG | 6 | 27764843 | ^56^ |
