# Supplementary material for: Modulating CRISPR-Cas Genome Editing Using Guide-Complementary DNA Oligonucleotides
Source: CRISPR J. 2022 Aug 12;5(4):571–85. doi: 10.1089/crispr.2022.0011 (PMC9419950; doi:10.1089/crispr.2022.0011)
Supplement: Supplemental data [file Suppl_TableS2.docx]

| **Supplementary table 2. Deep-sequencing preparation PCR conditions.**  **Left:** The components, their concentrations and used volumes for the 25µL PCR reactions.  **Right:** The thermocycler program used for the PCRs. | | | | | |
| --- | --- | --- | --- | --- | --- |
| **component** | **volume (ul)** |  | **temp. (°C)** | **duration** |  |
| 2x Q5 mastermix | 12.5 |  | 98 | 30s |  |
| fw primer 2.5µM | 5.0 |  | 98 | 10s | 25x |
| rv primer 25µM | 0.5 |  | (supp. table 2) | 30s |  |
| DNA 28ng/µL | 7 |  | 72 | 15s |  |
| **total** | **25** |  | 72 | 2min |  |
