# Supplementary material for: Modulating CRISPR-Cas Genome Editing Using Guide-Complementary DNA Oligonucleotides
Source: CRISPR J. 2022 Aug 12;5(4):571–85. doi: 10.1089/crispr.2022.0011 (PMC9419950; doi:10.1089/crispr.2022.0011)
Supplement: Supplemental data [file Suppl_TableS3.docx]

| **Supplementary table 3. Deep-sequencing preparation primers.**  The primers that were you to create the amplicons that were sent for deep-sequencing. In the sequences, ‘---‘ is used to indicate the upstream sequences that Baseclear B.V. requested for further preparation of the sequencing samples. These sequences are excluded from the amplicon length in the last column. ‘NNNNN’ is used to indicate the 5 nt long barcodes that we used to distinguish between conditions. | | |
| --- | --- | --- |
| **primer** | **sequence** | **annealing temperature** |
| EMX1-1_on_fw | ---NNNNNAAGGTGTGGTTCCAGAACC | 66°C |
| EMX1-1_on_rv | ---CGATGTCCTCCCCATTGG |  |
| EMX1-1_off-1_fw | ---NNNNNTTACCATAGACTATCACC | 54°C |
| EMX1-1_off-1_rv | ---GGTTACAGAAAGAATAGG |  |
| EMX1-1_off-2_fw | ---NNNNNTGTGCTTCAACCCATCACG | 67°C |
| EMX1-1_off-2_rv | ---GCAGTCTCATGACTTGGCC |  |
| FANCF-2_on_fw | ---NNNNNCGTAGGTAGTGCTTGAGACC | 65°C |
| FANCF-2_on_rv | ---CATTTCGCGGATGTTCCAATC |  |
| FANCF-2_off-1_fw | ---NNNNNCCATTTCTGTCTCCACCTCC | 62°C |
| FANCF-2_off-1_rv | ---CCTCTCTCTTCCACCGAGTTAC |  |
| FANCF-2_off-2_fw | ---NNNNNGTTTAATGTACAAGGGGTGAG | 61°C |
| FANCF-2_off-2_rv | ---CAATCCAGGGCCCTATCTC |  |
