# Supplementary material for: Modulating CRISPR-Cas Genome Editing Using Guide-Complementary DNA Oligonucleotides
Source: CRISPR J. 2022 Aug 12;5(4):571–85. doi: 10.1089/crispr.2022.0011 (PMC9419950; doi:10.1089/crispr.2022.0011)
Supplement: Supplemental data [file Suppl_TableS4.docx]

| **Supplementary table 4. *In vitro* DNA substrate primers**  The primers used to amplify the 1500bp DNA substrates from the human genome for the *in vitro* assays. Cleavage by Cas9 results in two fragments of lengths 500bp and 1000bp. | | |
| --- | --- | --- |
| **Locus** | **fw primer** | **rv primer** |
| EMX1-1 | tctcatttactactcacatccactctg | agggcttaaggctgagcc |
| FANCF-2 | cgctttacaggtctccagg | cctattaatgccaggcgctatg |
