# Supplementary material for: Modulating CRISPR-Cas Genome Editing Using Guide-Complementary DNA Oligonucleotides
Source: CRISPR J. 2022 Aug 12;5(4):571–85. doi: 10.1089/crispr.2022.0011 (PMC9419950; doi:10.1089/crispr.2022.0011)
Supplement: Supplemental data [file Suppl_FigS1.docx]

| **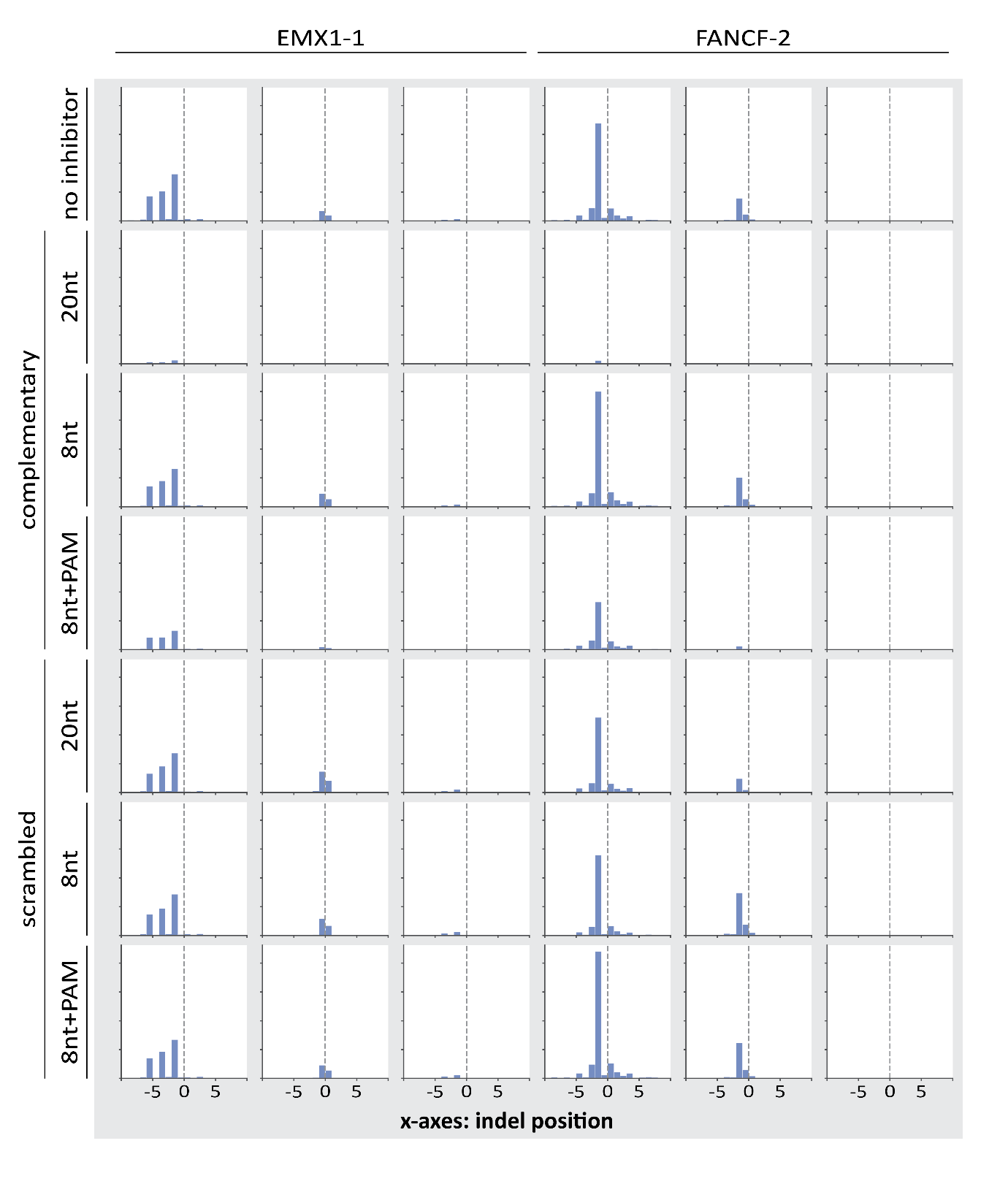** |
| --- |
| **Supplementary figure 1. Indel positions distribution.**  Histograms of the positions of the indels for different DNA oligo-based designs. The ‘0’ position is the Cas9 cut-site for each amplicon. We only displayed indels that occurred within 9 bp from the cut-site. The DNA oligos included in these graphs were delivered at molar concentrations equal to the concentration of Cas9 and guide RNA. |
