# Supplementary material for: Modulating CRISPR-Cas Genome Editing Using Guide-Complementary DNA Oligonucleotides
Source: CRISPR J. 2022 Aug 12;5(4):571–85. doi: 10.1089/crispr.2022.0011 (PMC9419950; doi:10.1089/crispr.2022.0011)
Supplement: Supplemental data [file Suppl_FigS2.docx]

| **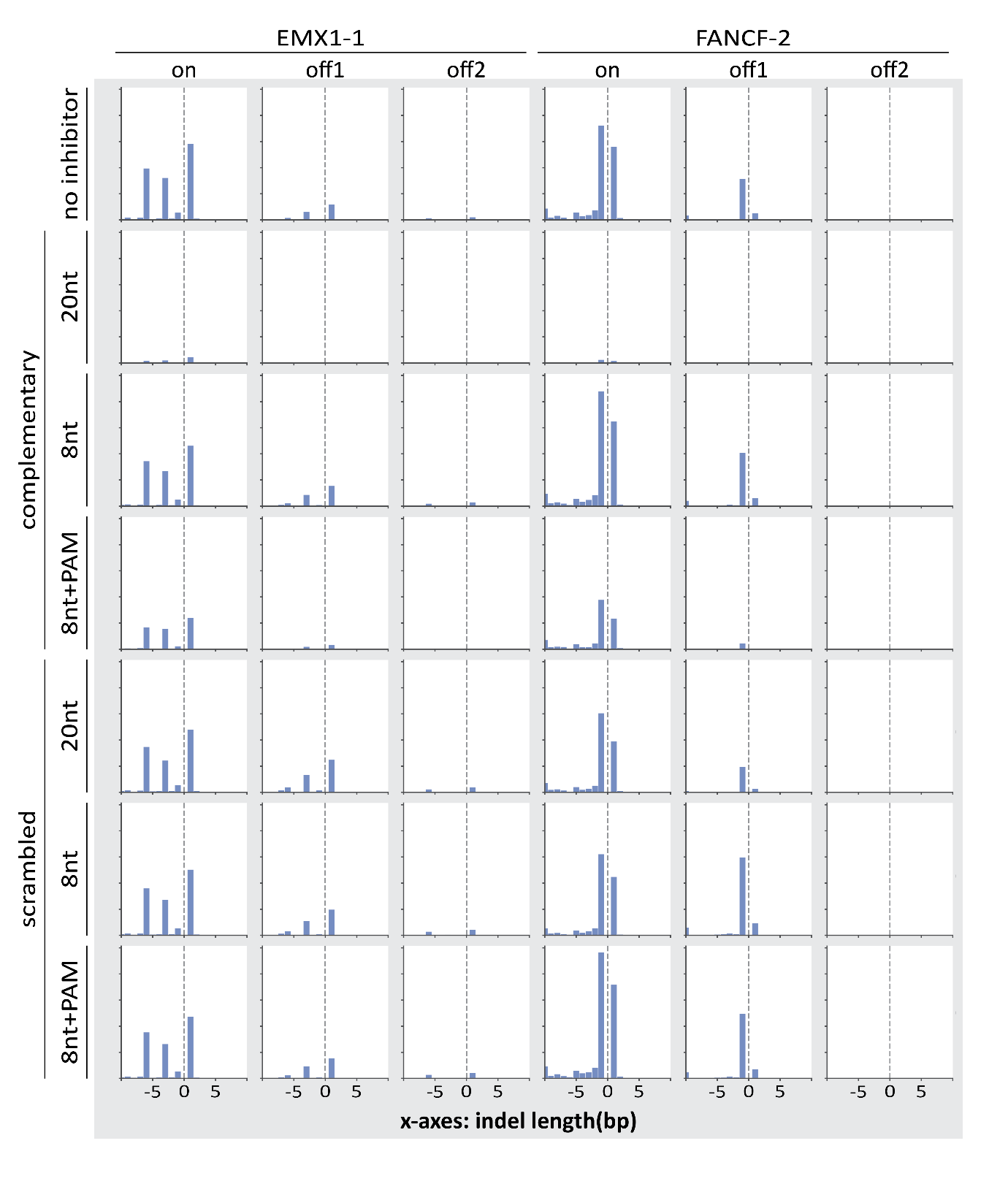** |
| --- |
| **Supplementary figure 2. Indel length distribution.**  Histograms of the lengths of the indels for different DNA oligo-based designs. Negative lengths indicate deletions, whereas positive lengths indicate insertions. A length of ‘0’ would not be an indels, and was not included in these graphs. We only displayed insertions or deletions or 10 bp or shorter. The DNA oligos included in these graphs were delivered at molar concentrations equal to the concentration of Cas9 and guide RNA. |
