# Supplementary material for: Modulating CRISPR-Cas Genome Editing Using Guide-Complementary DNA Oligonucleotides
Source: CRISPR J. 2022 Aug 12;5(4):571–85. doi: 10.1089/crispr.2022.0011 (PMC9419950; doi:10.1089/crispr.2022.0011)
Supplement: Supplemental data [file Suppl_FigS3.docx]

| 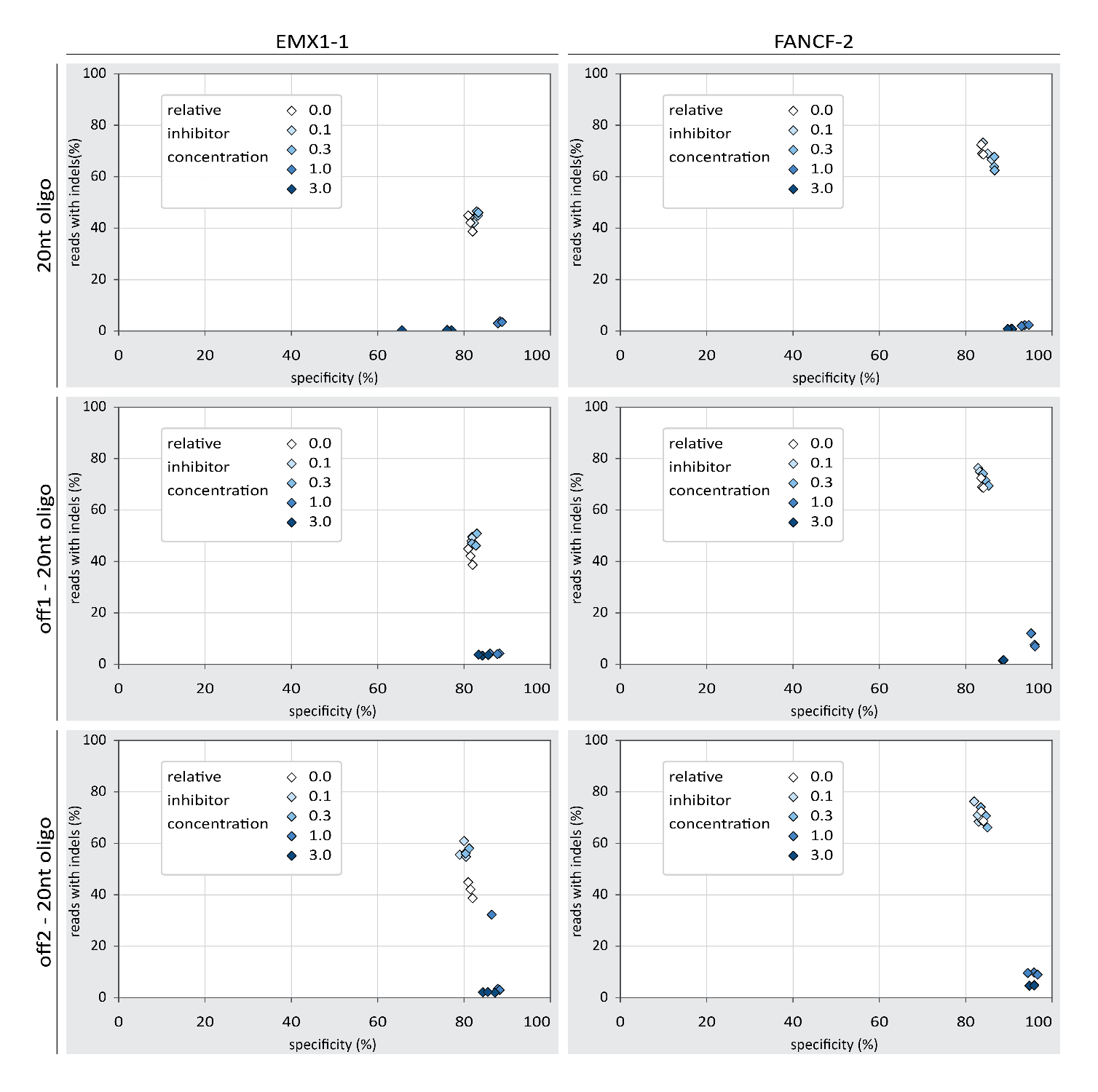 |
| --- |
| **Supplementary figure 3. 20nt and off-target complementary oligo activity and specificity.**  Comparison of percentage reads with indels and percentage specificity for different concentrations of the 20nt inhibitor. Also included variants of the 20nt inhibitor that are complementary to the off-targets (off1 and off2) instead of being complementary to the on-target. For each inhibitor concentration, individual replicates are displayed as diamonds with the same color. |
