# Supplementary material for: Modulating CRISPR-Cas Genome Editing Using Guide-Complementary DNA Oligonucleotides
Source: CRISPR J. 2022 Aug 12;5(4):571–85. doi: 10.1089/crispr.2022.0011 (PMC9419950; doi:10.1089/crispr.2022.0011)
Supplement: Supplemental data [file Suppl_TableS5.docx]

| **Supplementary table 5. DNA oligo-based inhibitors**  The names, sequences, and lengths of the DNA oligonucleotides that were used as inhibitors. In the sequences, both the guide-complementary part and the PAM are in uppercase, but the loop connecting them is lowercase. | | | |
| --- | --- | --- | --- |
| **site** | **inhibitor name** | **DNA oligo sequence** | **length (nt)** |
| **EMX1-1** | 8nt + PAM | AGGcaaaagcctTTCTTCTT | 20 |
|  | 20nt | TTCTTCTTCTGCTCGGACTC | 20 |
|  | off1 - 20nt | TTCTTCTTCTGCTCTAACTC | 20 |
|  | off2 - 20nt | TTCTTCTTCTGCTTAGACTC | 20 |
|  | 8nt | TTCTTCTT | 8 |
|  | scrambled 8nt + PAM | AGGcaaaagcctCTTTCTTT | 20 |
|  | scrambled 20nt | CTGTACGTGCCCTCTTTCTT | 20 |
|  | scrambled 8nt | CTTTCTTT | 8 |
|  | 8nt + PAM 1mm | AGGcaaaagcctTTCTTCTa | 20 |
|  | 8nt + PAM 2mm | AGGcaaaagcctTTCTTCga | 20 |
|  | 8nt + PAM 3mm | AGGcaaaagcctTTCTTtga | 20 |
|  | 8nt + PAM 4mm | AGGcaaaagcctTTCTgtga | 20 |
|  | 8nt + PAM 5mm | AGGcaaaagcctTTCggtga | 20 |
|  | 8nt + PAM 6mm | AGGcaaaagcctTTaggtga | 20 |
|  | 8nt + PAM 7mm | AGGcaaaagcctTgaggtga | 20 |
|  | 8nt + PAM 8mm | AGGcaaaagcctggaggtga | 20 |
| **FANCF-2** | 8nt + PAM | AGGcaaaagcctCATGGAAT | 20 |
|  | 20nt | CATGGAATCCCTTCTGCAGC | 20 |
|  | off1- 20nt | CTTGGAATCCCTTCTGCAGC | 20 |
|  | off2- 20nt | CATGGAGTCCCTTCTGCGTC | 20 |
|  | 8nt | CATGGAAT | 8 |
|  | scrambled 8nt + PAM | AGGcaaaagcctAGTCAAGT | 20 |
|  | scrambled 20nt | AGAATCCCACGCTTCCGTTG | 20 |
|  | scrambled 8nt | AGTCAAGT | 8 |
|  | 8nt + PAM 1mm | AGGcaaaagcctCATGGAAg | 20 |
|  | 8nt + PAM 2mm | AGGcaaaagcctCATGGAgg | 20 |
|  | 8nt + PAM 3mm | AGGcaaaagcctCATGGggg | 20 |
|  | 8nt + PAM 4mm | AGGcaaaagcctCATGtggg | 20 |
|  | 8nt + PAM 5mm | AGGcaaaagcctCATctggg | 20 |
|  | 8nt + PAM 6mm | AGGcaaaagcctCAcctggg | 20 |
|  | 8nt + PAM 7mm | AGGcaaaagcctCtcctggg | 20 |
|  | 8nt + PAM 8mm | AGGcaaaagcctatcctggg | 20 |
