# Supplementary material for: Modulating CRISPR-Cas Genome Editing Using Guide-Complementary DNA Oligonucleotides
Source: CRISPR J. 2022 Aug 12;5(4):571–85. doi: 10.1089/crispr.2022.0011 (PMC9419950; doi:10.1089/crispr.2022.0011)
Supplement: Supplemental data [file Suppl_FigS5.docx]

| **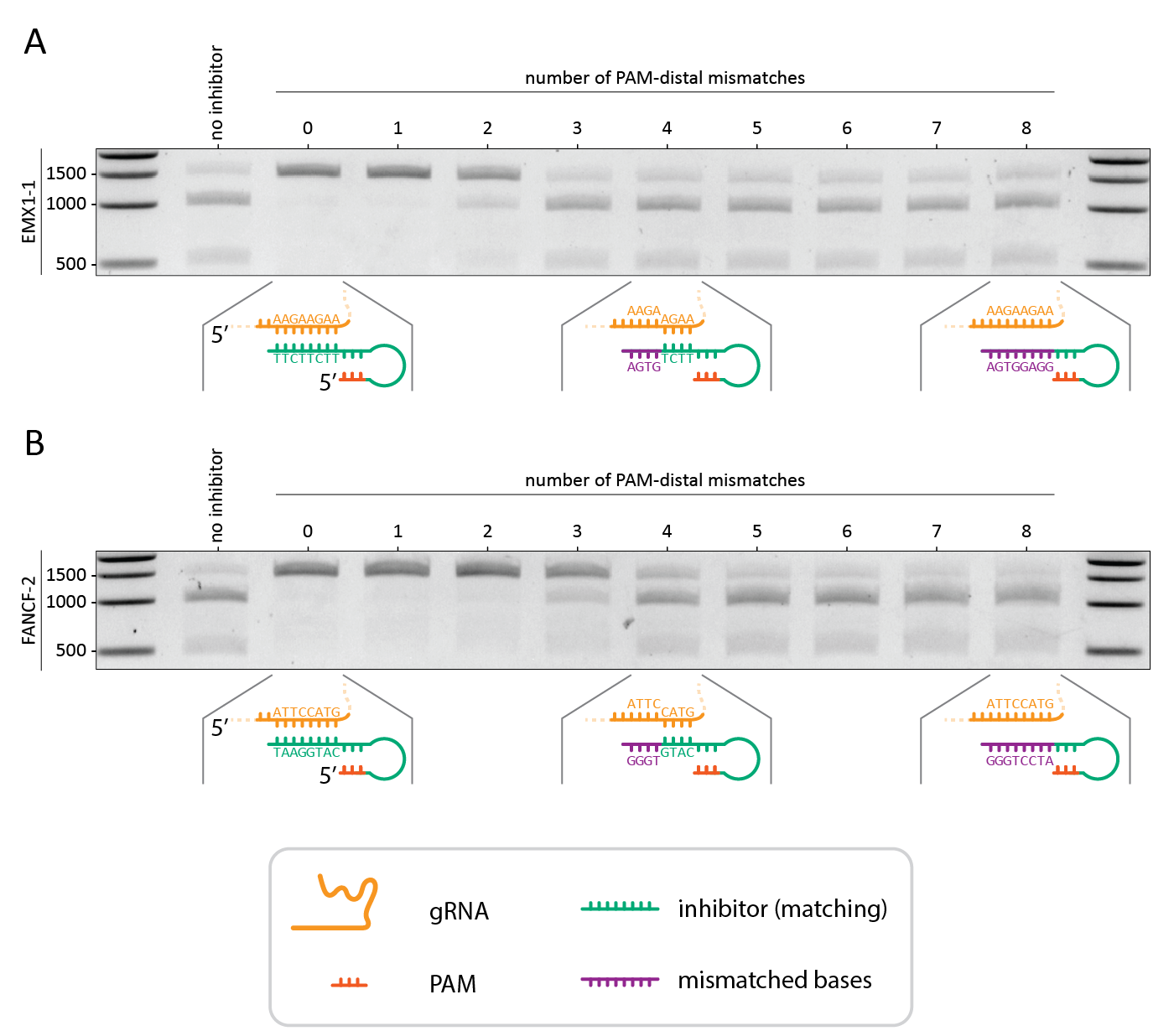** |
| --- |
| **Supplementary figure 5. Sequence complementarity requirement**  Agarose gel images from an *in vitro* assay with 8nt+PAM inhibitors containing increasing numbers of PAM distal mismatches. The inhibitors were added to pre-formed RNP complexes as in the ‘Cas9 + gRNA pre-incubation’ samples in Figure 5 of the main text. Below each gel, a schematic is used to illustrate the design of the inhibitors with 0, 4, and 8 mismatches compared to the guide RNA. The 1kb ladder (New England Biolabs) was included in the outer most lanes of the gel and relevant fragment lengths are indicated in base-pairs. The linear substrate DNA is 1500bp long and cleavage by Cas9 would result in 2 fragments of lengths 1000bp and 500bp. The images shown are from the first of two replicates that were conducted, which both gave the same results. |
