# Supplementary material for: Modulating CRISPR-Cas Genome Editing Using Guide-Complementary DNA Oligonucleotides
Source: CRISPR J. 2022 Aug 12;5(4):571–85. doi: 10.1089/crispr.2022.0011 (PMC9419950; doi:10.1089/crispr.2022.0011)
Supplement: Supplemental data [file Suppl_FigS6.docx]

| **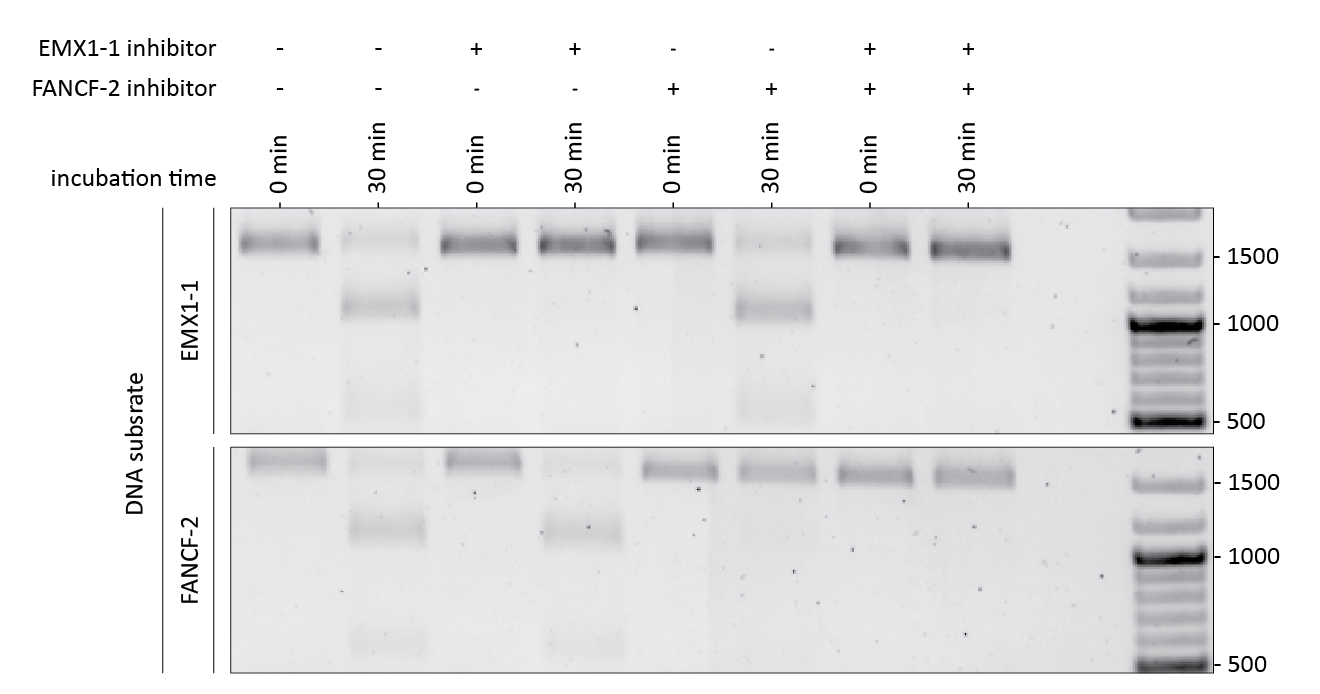** |
| --- |
| **Supplementary figure 6. Independent inhibition of Cas9-guide combinations**  Agarose gel images from an *in vitro* cleavage assay with oligo-based inhibitors that selectively inhibit one combination of Cas9 and guide while leaving the other guide unaffected. In all conditions, guides were present for both EMX1-1 and FANCF-2. Different combinations of 8nt+PAM inhibitors were added as displayed above the gel (the inhibitor(s) with a ‘+’ sign were present, whereas those with a ‘-‘ sign were not). Either EMX1-1 or FANCF-2 DNA substrate was added as indicated on the left. For each condition, samples were loaded from 0, or 30 minutes of incubation for DNA cleavage. For the 0 minute samples, no cleavage is expected or observed in any of the conditions. This experiment was conducted with two RNP concentrations: one concentration where each RNP is at the same concentration as used in the other *in vitro* assays and another where the total RNP concentration is equal to the other *in vitro* assays. Only the latter is shown here but both gave the same results. The Generuler mix ladder was included in the right most lanes of the gels and relevant fragment lengths are indicated in base-pairs. The linear substrate DNA is 1500bp long and cleavage by Cas9 would result in 2 fragments of lengths 1000bp and 500bp. The images shown are from the first of two replicates that were conducted, which both gave the same results. |
