# Supplementary material for: Modulating CRISPR-Cas Genome Editing Using Guide-Complementary DNA Oligonucleotides
Source: CRISPR J. 2022 Aug 12;5(4):571–85. doi: 10.1089/crispr.2022.0011 (PMC9419950; doi:10.1089/crispr.2022.0011)
Supplement: Supplemental data [file Suppl_FigS4.docx]

| 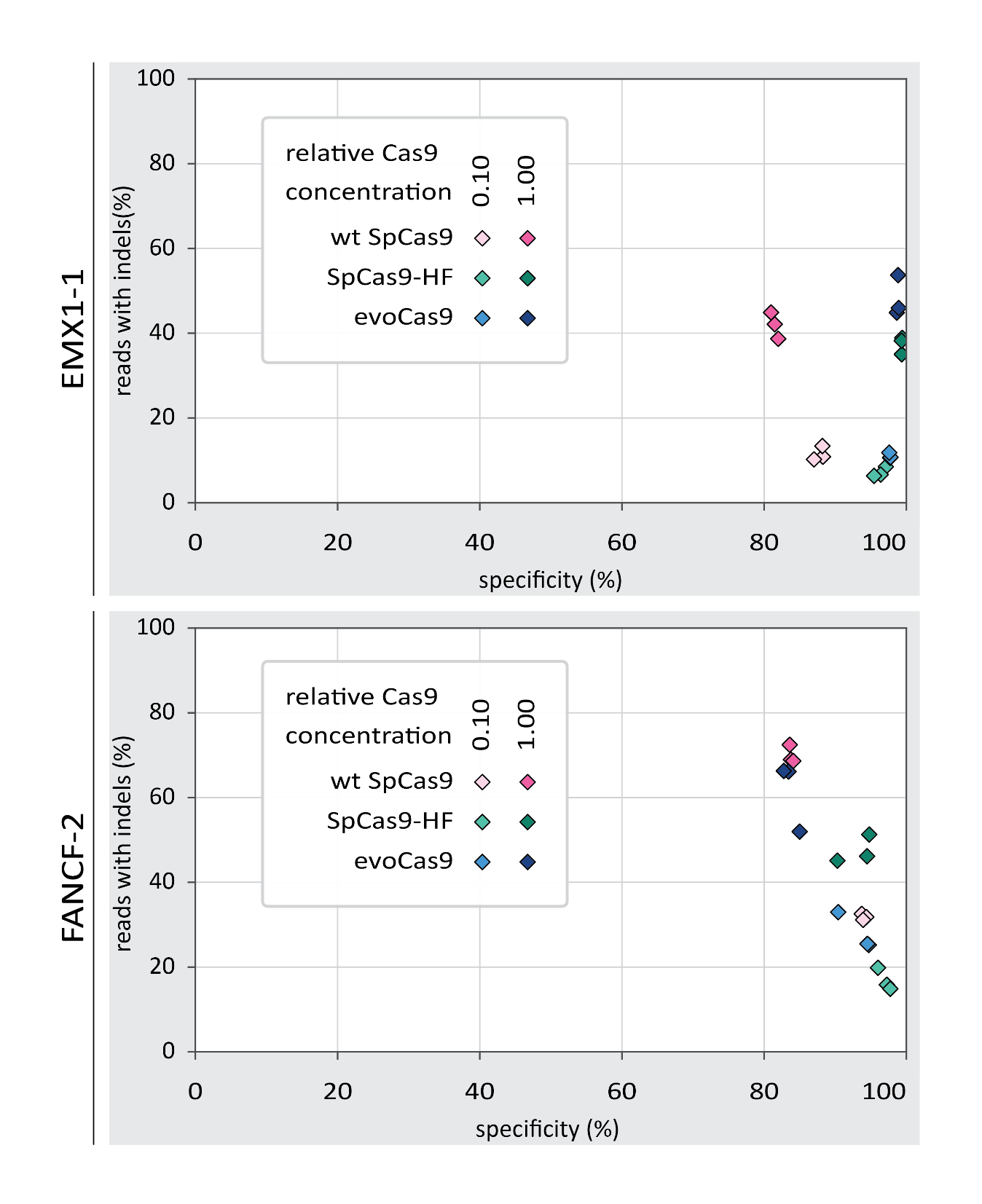 |
| --- |
| **Supplementary figure 4. Specificity and activity of engineered Cas9 variants.**  Comparison of *percentage reads with indels* and *percentage specificity* for different Cas9 variants. For each protein, and for each protein concentration, individual replicates are displayed as diamonds with the same color. |
